# Supplementary material for: Spatial Analysis of Schistosomiasis in Hunan and Jiangxi Provinces in the People’s Republic of China
Source: Diseases. 2022 Oct 19;10(4):93. doi: 10.3390/diseases10040093 (PMC9590053; doi:10.3390/diseases10040093)
Supplement: Supplementary file 1 [file diseases-10-00093-s001.zip › diseases-1882794-supplementary.pdf]

Table S1: Data sources and definitions of covariates

Covariates	Data sources	Definitions
Distance to water body	Global Lakes and Wetlands Database (GLWD)	Distance to permanent and semi-permanent water based on presence of lakes, wetlands, rivers and streams, and accounting for slope and precipitation (36)
Access to healthcare facilities	Malaria Atlas Project (MAP)	Walking travel times in minutes to the nearest health facilities (22)
Temperature	Global climate data	Annual mean environmental air temperature (°C) (19)
Precipitation	Global climate data	Annual mean rainfall (mm) (19)
Solar radiation	Global climate data	Annual mean solar radiation (kJ m-2 day-1) (19)
Altitude	Shuttle Radar Topography Mission (SRTM)	Elevation of the earth land surface in km (37)

Table S2: Watanabe-Akaike information criterion (WAIC) values corresponding to different model specifications.

Model specifications	WAIC	
	Hunan	Jiangxi
Distance to water body	87.7	64.4
Distance to water body + distance to health facilities	89.4	64.9
Distance to water body + distance to health facilities + altitude	91.5	62.3
Distance to water body + distance to health facilities + altitude + temperature	90.9	58.9

Precipitation and sun radiation were removed from the model due to multicollinearity.

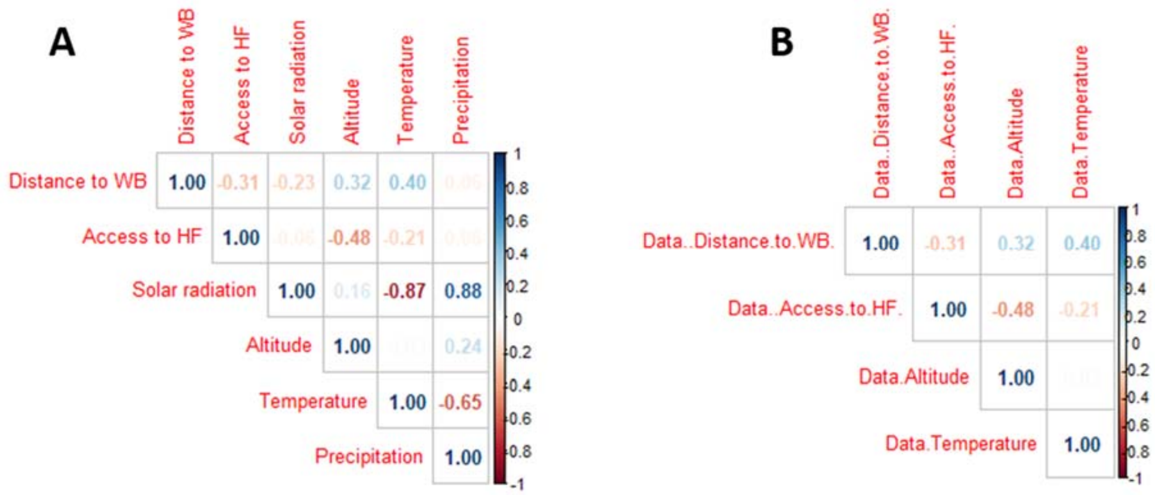

Figure S1: Multicollinearity for all variables (A) and for selected variables for the final model (B). There was multicollinearity for precipitation and sun radiation and these variables were removed from the mode. After removing precipitation and sun radiation, multicollinearity has not been observed in the reaming variables.

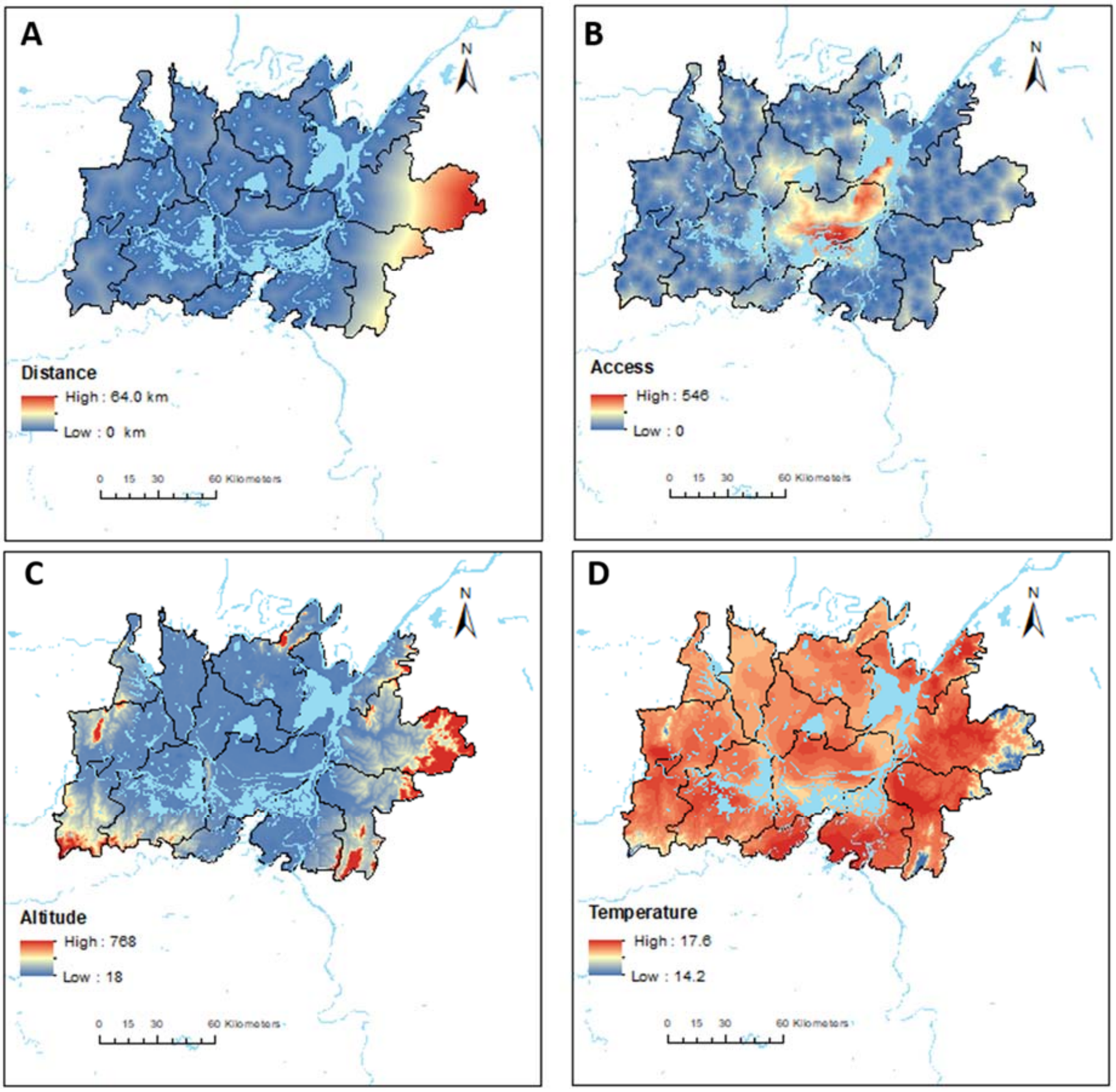

Figure S2: Maps showing the distribution of covariates fitted to the model in Hunan Province: (A) distance to waterbody (km), (B) access to healthcare facilities (walking travel times in min), (C) altitude (km), (D) temperature (°C).

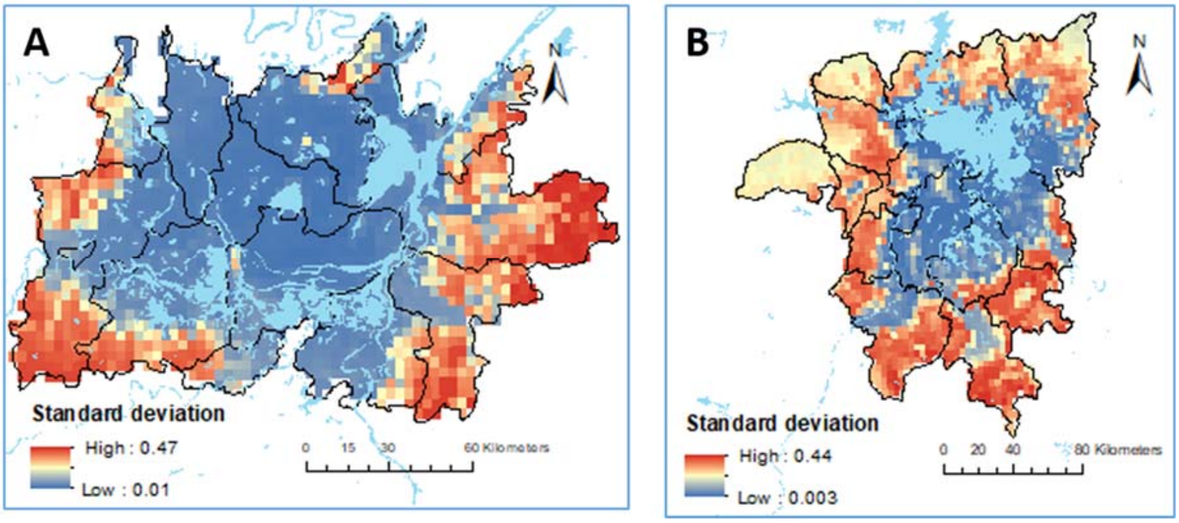

Figure S3: The standard deviation of the predicted prevalence of Schistosoma in Hunan (A) and Jiangxi (B).

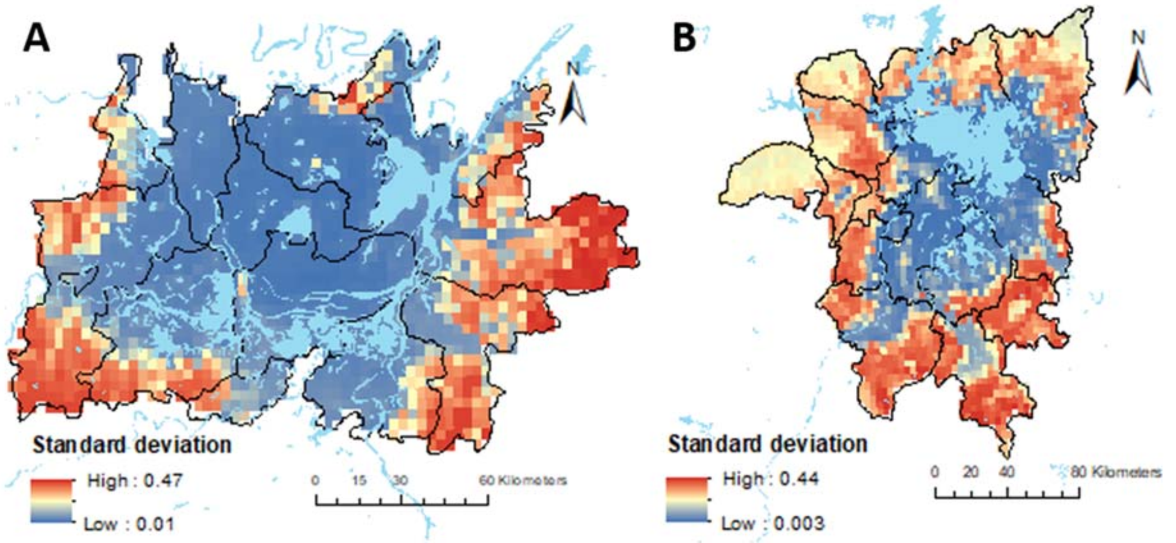

Figure S4: The standard deviation of the predicted prevalence of Schistosoma in Hunan (A) and Jiangxi (B).
